# The effects of different cold‐temperature regimes on development, growth, and susceptibility to an abiotic and biotic stressor

**DOI:** 10.1002/ece3.4957

**Published:** 2019-02-20

**Authors:** Matthew Wersebe, Paradyse Blackwood, Ying Tong Guo, Jared Jaeger, Dyllan May, George Meindl, Sean N. Ryan, Vivian Wong, Jessica Hua

**Affiliations:** ^1^ Biological Sciences Department Binghamton University (SUNY) Binghamton New York

**Keywords:** climate change, Echinostomidae, *Lithobates sylvaticus*, secondary salinization

## Abstract

1. Global climate change is expected to both increase average temperatures as well as temperature variability.
2. Increased average temperatures have led to earlier breeding in many spring‐breeding organisms. However, individuals breeding earlier will also face increased temperature fluctuations, including exposure to potentially harmful cold‐temperature regimes during early developmental stages.
3. Using a model spring‐breeding amphibian, we investigated how embryonic exposure to different cold‐temperature regimes (control, cold‐pulse, and cold‐press) affected (a) compensatory larval development and growth, (b) larval susceptibility to a common contaminant, and (c) larval susceptibility to parasites.
4. We found: (a) no evidence of compensatory development or growth, (b) larvae exposed to the cold‐press treatment were more susceptible to NaCl at 4‐days post‐hatching but recovered by 17‐days post‐hatching, and (c) larvae exposed to both cold treatments were less susceptible to parasites.
5. These results demonstrate that variation in cold‐temperature regimes can lead to unique direct and indirect effects on larval growth, development, and response to stressors. This underscores the importance of considering cold‐temperature variability and not just increased average temperatures when examining the impacts of climate disruption.

## 1 INTRODUCTION

Increases in average global temperatures and heightened temperature variability are both consequences of global climate change (IPCC, 2014, 2018). A commonly documented ecological consequence of increasing average temperatures is shifts in phenological patterns (i.e., the movement of event times‐ breeding, flowering, mortality; Walther et al., 2002; Parmesan & Yohe, 2003; Thackeray et al., 2016). In particular, warming average temperatures are associated with early‐onset spring conditions and in many taxa, a subsequent acceleration in key life history traits, such as breeding dates (Li, Cohen, & Rohr, 2013; Walther et al., 2002). Organisms that breed earlier in response to rising average spring temperatures also face heightened fluctuations in temperatures, including increased risk of exposure to harmful cold‐temperature regimes during early developmental stages (Benard, 2015; Cohen, Pfeiffer, & Francis, 2018; Inouye, 2008; Kretschmer et al., 2017). For example, in plants, earlier breeding triggered by warmer winters led to exposure to increasing variance in temperature, paradoxically including increased risk of frost damage (Inouye, 2008). Therefore, evaluating the consequences of different temperature regimes (i.e., cold‐temperature variability) and not just increasing average temperatures is crucial to understanding the ecological impact of global climate change.

Cold‐temperature conditions can have diverse direct and indirect consequences. For example, cold temperatures can slow metabolic rate, development, and reduce decomposition, digestion, growth, swimming, and feeding activity (Brown, Gillooly, Allen, Savage, & West, 2004; Podolsky & Emlet, 1993; Yee & Murray, 2004). Cold temperatures may also have indirect consequences by creating mismatches in resource availability for organisms with advanced spring phenology (e.g., food and habitat; Visser & Both, 2005). Despite the negative consequences of cold temperatures, many organisms, especially those historically exposed to predictably low temperatures, can compensate for negative effects both through behavior and physiology. For example, rainbow trout exposed to cold temperatures have decreased visceral adipose tissue and lipid content, but over time affected trout display compensatory growth to maintain a body length comparable to those reared in optimal temperatures (Weatherley & Gill, 1983). Although a diverse range of taxa can produce compensatory growth and developmental responses to low temperatures, the cold‐temperature regimes that organisms encounter are unlikely to be uniform. For example, organisms may be exposed to prolonged cold‐conditions (i.e., cold‐press) or intermittent cold‐conditions (i.e., cold‐pulse) across time. Whether organisms respond uniformly to different cold‐temperature regimes is not well understood but has broad ecological and conservation implications for our understanding of the impact of global climate change.

While many organisms respond to cold temperatures by inducing compensatory growth and development, these compensatory responses may also come at a cost in response to other stressors in the environment. For instance, along with temperature shifts associated with global climate change, natural ecosystems face a variety of other abiotic threats including anthropogenic contaminants (Boone et al., 2014; Dugan et al., 2017) that can interact with shifts in temperature to more negatively affect organisms. For example, silver perch reared in higher temperatures (30 and 35°C) were more susceptible to the insecticide endosulfan than silver perch reared at lower temperatures (15–25°C; Patra, Chapman, Lim, Gehrke, & Sunderam, 2015). Related to cold weather regimes, to combat icy road conditions, road salt application has increased from 5,000 tons in 1941 to between 10 and 20 million tons in 2010, leading to consequences such as the secondary salinization of freshwater habitats (Kelly, Findlay, Schlesinger, Menking, & Chatrchyan, 2010). Thus, the likelihood of exposure to both cold‐temperature regimes and contaminants such as road salt are high during early spring months (Dietz, Angel, Robbins, & McNaboe, 2016). However, to date, it is unclear how exposure to cold‐temperature regimes early in development may influence responses to contaminants, such as salt, later in life.

In addition to abiotic stressors, organisms also face a variety of biotic stressors such as infectious diseases (Daszak, Cunningham, & Hyatt, 2000). A growing number of studies demonstrate that shifting temperatures can lead to alterations in host–parasite interactions (Brooks & Hoberg, 2007, 2015; Kutz, Hoberg, Polley, & Jenkins, 2005). For example, an increase in temperature variability can increase amphibian susceptibility to infection by parasites such as *Batrachochytrium dendrobatidis* (Raffel, Rohr, Kiesecker, & Hudson, 2006; Raffel et al., 2013; Rohr & Raffel, 2010). Similarly, in amphipods, the ability to clear bacterial infections was negatively affected when reared in low and high temperatures relative to intermediate temperatures (Labaude, Moret, Cézilly, Reuland, & Rigaud, 2017). While warming and variable warm temperatures have been shown to alter susceptibility to host–parasite interactions (Rumschlag, Boone, & Fellers, 2014), the effects of exposure to cold and variable cold temperatures on disease susceptibility are less understood (Cohen et al., 2017; Raffel et al., 2013; Rohr & Raffel, 2010). Understanding the cost of compensatory responses not just on growth and development but on responses to other abiotic and biotic stressors in the environment is important to evaluate the effects of global climate change on ecological systems (Carey & Alexander, 2003).

Toward these goals, using an amphibian model, we ask: how does embryonic exposure to varying cold‐temperature regimes (cold‐pulse vs. cold‐press) affect (a) tadpole development and growth across time, (b) tadpole susceptibility to a common contaminant (NaCl), and (c) tadpole susceptibility to a common parasite (trematode)? We hypothesized that embryonic exposure to both cold‐temperature regimes would lead to an initial reduction in tadpole development and growth. However, over time, we predicted that tadpoles from both cold treatments would display compensatory development and growth thereby reducing or eliminating the negative effects of the cold treatments on development and growth. Similarly, we predicted that embryonic exposure to cold‐temperature regimes would be costly and would negatively affect the ability for tadpoles to respond to both NaCl and parasite stressors later in life.

## 2 METHODS

### 2.1 Model system

We chose *Lithobates sylvaticus *(wood frog) as the focal species for examining the impacts of different cold‐temperature regimes (cold‐pulse and cold‐press). Wood frogs breed in small woodland ponds, are among the most widely distributed and abundant anurans in North America (Conant & Collins, 1998), and are recognized as important faunal indicators of ecosystem health (Hilty & Merenlender, 2000). Wood frogs range from northern subarctic Canada and Alaska to the Northeast and Midwestern United States (Conant & Collins, 1998). Wood frogs exhibit a temperature‐dependent induction of breeding behavior and depending on geographic location begin breeding in March through June (Frisbie, Costanzo, & Lee, 2000; Herreid & Kinney, 1967; Howard, 1980; Pollister & Moore, 1937). Wood frogs are under strong selection for early oviposition (Howard, 1980) as early oviposition is related with central egg mass positioning within the greater egg mass cluster, which is associated with higher hatching success and survivorship (Waldman, 1982). Compared to other amphibians, Gibbs and Breisch (2001) found that wood frogs show one of the strongest shifts (13.0 days per century) to earlier calling and breeding phenology in response to recent climate warming. Furthermore, Benard (2015) found that offspring of wood frog populations that breed earlier experienced colder temperatures throughout development. Thus, wood frogs are a good model for understanding responses to cold‐temperature regimes and costs associated with these responses.

To evaluate costs of responses to different cold‐temperature regimes, we first assessed tadpole susceptibility to NaCl. The secondary salinization of freshwater habitats due to human activities (i.e., road salts, agriculture, coastal flooding) is of emerging concern among ecologists and natural resource managers (Cañedo‐Argüelles et al., 2016; Herbert et al., 2015). We chose NaCl because the leading cause of freshwater salinization within the range of our focal species (wood frog) is the application of road deicing salts (primarily NaCl; Findlay & Kelly, 2011; Kaushal et al., 2005; Kelly et al., 2010). Further, the timing of peak chloride concentrations via snowmelt runoff coincides directly with the breeding and larval period of wood frogs (Findlay & Kelly, 2011; Sanzo & Hecnar, 2006). Acute salt tolerance assays suggest that wood frogs can tolerate between 2.6 g/L and 17.2 g/L Cl^−^ (Collins & Russell, 2009; Sanzo & Hecnar, 2006) but studies show that increasing salinity is extremely impactful to population demography, possibly leading to exclusion from roadside breeding sites and local extinction (Collins & Russell, 2009; Karraker, Gibbs, & Vonesh, 2008).

Next, we assessed how responses to different cold‐temperature regimes affected tadpole susceptibility to a common amphibian trematode in the Echinostomatidae family. Trematodes have a complex seven‐part life cycle: (a) The adult form of the parasite reproduces sexually and produce eggs in the digestive tract of a mammalian or avian definitive host. (b) The definitive host excretes the eggs into aquatic environment. (c) A free‐swimming stage of the parasite, miracidia, emerges from the egg and infects molluscan hosts, the first intermediate host. (d) Within the molluscan host, the parasite develops and multiplies asexually. (e) The parasite then emerges in its second free‐swimming stage, cercariae. (f) Cercariae encyst in the kidneys of larval amphibians, the second intermediate host forming metacercariae. (g) The intermediate host and embedded metacercariae are consumed by a mammalian or avian host where the cycle begins again (Esteban & Muñoz‐Antoli, 2009). Metacercariae from the Echinostomatidae family are reported as one of the most common parasites in larval amphibians (Maldonado & Lanfredi, 2009) and exhibit a dose‐dependent pathology (Johnson & McKenzie, 2009; Martin & Conn, 1990). In this study, we focus on the cercariae stage of the parasite as this is the stage that infects larval amphibians. Trematode susceptibility in larval amphibians is stage and life history dependent (Holland et al., 2007; Johnson et al., 2012); thus alterations to the rate of tadpole development due to cold temperatures may have significant implications for the outcome of this host–parasite interaction.

### 2.2 Animal collection

On April 5, 2017, we collected 10 newly laid wood frog egg masses from Lipo Pond (42°3′45.12″N, 76°3′47.66″W; Vestal, New York). We immediately transferred all egg masses to Binghamton University where we placed them in 100 L outdoor pools filled with 90 L of well water. On April 6, 2017, we separated 18 individual eggs from each of the 10 egg masses for a total of 180 eggs using a plastic transfer pipette. Then, we placed each egg into individual 20 ml scintillation vials containing 15‐ml UV‐filtered well water. All applicable institutional and/or national guidelines for the care and use of animals were followed (IACUC protocol #757‐16).

### 2.3 Experimental conditions

**Figure 1:**
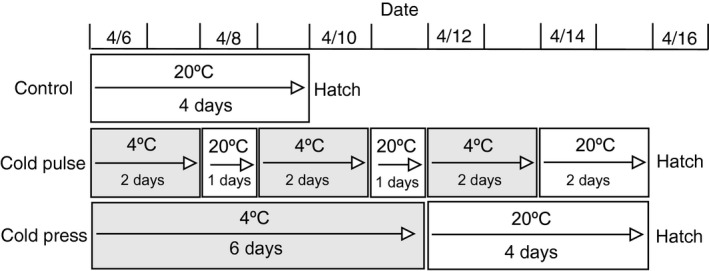
Schedule of embryonic exposure (Gosner 8–20) to the three temperature regime treatments (control, cold‐pulse, and cold‐press) and timing of hatching for animals from each treatment

On April 6, 2017, we randomly assigned wood frog embryos (Gosner stage 8; Gosner, 1960) to one of the three temperature regimes: 20°C (control; *n* = 60), 4°C/20°C (cold‐pulse; *n* = 60), and 4°C (cold‐press; *n* = 60; Figure 1). All embryos were held on a constant 12:12 light cycle. For all three treatments, we also held the amount of time that embryos were exposed to 20°C constant (4‐days; Figure 1; Table 1). For the two cold treatments (pulse and press), we manipulated the temperature regime, but we held the amount of time that embryos were exposed to 4°C constant (6 days; Figure 1; Table 1).

**Table 1:** Date and number of days post‐hatching for the following events: (a) hatching (Gosner 20), (b) Experiment 1: Time‐to‐death assays (c) Experiment 2: Parasite exposure for each of the three temperature regime treatments

Cold‐temperature regime	Event	Date	# days post‐hatch
Control	Hatched (Gosner 20)	April 9	0
	Experiment 1: Time‐to‐death assay		
	Gosner 25	April 13	4
	Gosner 31	April 26	17
	Experiment 2: Parasite exposure	May 3	24
Cold‐pulse	Hatched (Gosner 20)	April 15	0
	Experiment 1: Time‐to‐death assay		
	Gosner 25	April 19	4
	Gosner 31	May 2	17
	Experiment 2: Parasite exposure	May 9	24
Cold‐press	Hatched (Gosner 20)	April 15	0
	Experiment 1: Time‐to‐death assay		
	Gosner 25	April 19	4
	Gosner 31	May 2	1
	Experiment 2: Parasite exposure	May 9	24

In nature, wood frog embryos are exposed to a wide range of air and water temperatures (−12.3°C–26°C and 0.4°C–19.7°C, respectively; Frisbie et al., 2000; Benard, 2015; Hua et al., 2015a). Additionally, the lower developmental limit for wood frogs is reported to be between 2°C and 5.6°C (Herreid & Kinney, 1967; Pollister & Moore, 1937). Therefore, 4°C represents a temperature that is in between the reported lower developmental limits for wood frogs and both 4°C and 20°C represent realistic temperature experienced by wood frog embryos in nature.

#### 2.3.1 Embryonic control conditions

Individuals in the control treatment were kept in a temperature‐controlled room set at 20°C throughout development. On April 9, all individuals in the control treatment hatched (4‐days; Figure 1; Table 1). For the control treatment, all 60 embryos successfully hatched.

#### 2.3.2 Embryonic cold‐pulse conditions

We placed individuals in the cold‐pulse treatment in a temperature‐controlled room set at 4°C for 48 hr then moved them to a temperature‐controlled room set at 20°C for 24 hr. We replicated this pulse schedule for two more cycles resulting in a total of 6 days at 4°C and four days at 20°C. Individuals hatched at 20°C on April 15 (Figure 1; Table 1). For the cold‐pulse treatment, 46 embryos successfully hatched.

#### 2.3.3 Embryonic cold‐press conditions

Individuals in the cold‐press treatment were placed in a temperature‐controlled room set at 4°C for six days. Then, we transferred these individuals to a temperature‐controlled room set at 20°C until hatching. At 20°C, animals from the cold‐press treatment hatched in 4‐days on April 15 (Figure 1; Table 1). For the cold‐press treatment, 49 embryos successfully hatched.

After reaching the hatchling stage, we moved individuals from all temperature regime treatments to 1‐L plastic containers filled with 750 ml of UV‐filtered well water. We held all animals in a temperature‐controlled room set at 20°C until the start of Experiment 1 (time‐to‐death assay) or Experiment 2 (parasite exposure). Once individuals reached Gosner stage 25, we conducted water changes every 5 days and fed tadpoles a Tetramin slurry ad libitum.

### 2.4 Experiment 1: Time‐to‐death assay

We measured tadpole susceptibility to NaCl at two time points (4‐days post‐hatching and 17‐days post‐hatching) by conducting six separate (two for each cold‐temperature regime), but identical time‐to‐death assays (Newman, 2006). We conducted separate experiments to account for tadpoles from the different temperature regimes hatching on different days (Figure 1). For each time‐to‐death assay, we exposed 10 individuals from each temperature treatment to a lethal NaCl solution (10 g/L NaCl) and 5 individuals to UV‐filtered well water (control). Experimental units were 1‐L plastic containers containing 750‐ml of NaCl solution. We determined the time‐to‐death of each tadpole by assessing survival every four hours until 100% mortality. We preserved all individuals in a 10% formalin solution at the end of the experiment.

### 2.5 Experiment 2: Tadpole susceptibility to parasites 24‐days post‐hatching

We conducted three separate but identical parasite assays assessing the effects of the different cold‐temperature regimes on tadpole susceptibility to parasites. We conducted three separate experiments to account for tadpoles from the different temperature regimes hatching on different days (Figure 1). For each experiment, we randomly chose and exposed 10 individuals (24‐days post‐hatching) from each temperature treatment to 50 free‐swimming parasites. We also assigned five individuals from each of the three cold‐temperature regimes to serve as a no‐parasite control. Experimental units were 1‐L plastic containers containing 750‐ml of UV‐filtered well water.

To obtain parasites for these experiments, on April 23, we collected 30 ramshorn snails (*Helisoma trivolvis*) from the Binghamton University Nature Preserve wetland. We screened these snails for parasite infection by placing an individual snail in a 50 ml Falcon tube filled with 35 ml of UV‐filtered well water. Once we identified infected snails, we held snails at 2°C to slow parasite shedding until the start of each experiment (Hua, Buss, Kim, Orlofske, & Hoverman, 2016). For all three experiments, we used parasites shed from the same group of infected snails. Parasites were identified to family level using exposure assays and morphological observations (Buss & Hua, 2018; Kostadinova & Gibson, 2000). Prior to the start of each experiment, we acclimated snails at 20°C for 24 hr. Then snails were put into 50 ml Falcon tubes containing 45 ml of well water and placed under a 150‐watt heat lamp (EXO TERRA Solar Glo) to induce cercariae shedding. After shedding cercariae from each snail, we mixed cercariae from each tube to maximize genetic variation of cercariae as well as to avoid any bias from any single snail host. Using a glass pipette and stereo microscope, we individually counted and placed 50 cercariae into 30 Falcon tubes filled with 25 ml water (1,500 cercariae total). We then added 50 cercariae to each experimental unit by pouring the contents of each Falcon tube into units containing tadpole hosts and rinsing tubes out twice with the water from each unit to ensure that all cercariae were added. For the tadpoles assigned to the parasite‐control treatment, we mock dosed tadpoles using similar procedures but with 25 ml of parasite‐free water.

Twenty‐four hours after exposure to parasites, we euthanized (5 g/L MS‐222 solution) and preserved all tadpoles in 10% formalin solution and measured mass, stage (Gosner, 1960), and snout‐vent–length of all tadpoles. To quantify parasite infection, we removed the kidneys from each tadpole. We then placed the kidneys between microscopes slides and counted the number of encysted parasites using a stereo microscope (Olympus SZ60; Buss & Hua, 2018).

### 2.6 The effect of cold‐temperature regimes on development and growth

To assess the effect of cold‐temperature regimes on tadpole development and growth, we measured mass, stage (Gosner, 1960), and snout‐vent–length of tadpoles from each treatment at 4‐days, 17‐days, and 24‐days post‐hatching. It is important to note that we measured the development and mass of tadpoles using tadpoles from Experiment 1 and 2. Because all tadpoles were measured at the end of Experiment 1 and 2, tadpoles used for development and mass measurements were exposed to a lethal concentration of NaCl at 4‐days and 17‐days post‐hatching and 50 trematodes at 24‐days post‐hatching. Therefore, it is possible that development and growth may be affected by NaCl or trematode treatments. As such, it is not possible to compare development and mass across time points because metrics may be affected by NaCl or trematode exposure. However, because all tadpoles within a time point were similarly exposed to either NaCl or trematodes, we assessed development and mass within each time point.

### 2.7 Statistical analysis

#### 2.7.1 Effects of thermal regime on tadpole development and mass

To understand the effect of embryonic cold‐temperature regimes on tadpole development and mass 4‐days, 17‐days, and 24‐days post‐hatching, we conducted analysis of variance (ANOVA). All tadpoles at 4‐days and 17‐days post‐hatching were at the similar developmental stage 25 ± 0 and stage 31.4 ± 0.1, respectively; therefore, we did not conduct ANOVAs on tadpole mass during these time points. At 24‐days post‐hatching, tadpoles varied in both mass and development, so we conducted a multivariate analysis (MANOVA) that included both mass and developmental stage. We accounted for assumptions in the analysis and conducted pairwise comparisons for significant main effects. We compared the effects of the two cold treatments relative to the control using Dunnett's pairwise comparisons.

#### 2.7.2 Effects of cold‐temperature regime on tadpole susceptibility to NaCl

To investigate the effect of embryonic cold‐temperature regime on tadpole susceptibility to NaCl 4‐days and 17‐days post‐hatching, we conducted two separate generalized linear models with a normal distribution and an identity‐link function and incorporated tadpole mass as a covariate (GLM; McCullagh & Nelder, 1989). For all significant main effects, we conducted pairwise comparisons using Sequential Bonferroni‐corrected analyses.

#### 2.7.3 Effects of cold‐temperature regime on tadpole susceptibility to parasites 24‐days post‐hatching

To investigate the effect of embryonic cold‐temperature regime on tadpole susceptibility to parasites, we used a GLM with a Poisson‐distribution and a log‐link function because we had count data. For this analysis, we incorporated tadpole stage and mass as covariates as both have been shown to influence tadpole susceptibility to parasites (Johnson et al., 2012; Rohr, Raffel, & Hall, 2010). For all significant main effects, we conducted pairwise comparisons using sequential Bonferroni‐corrected analyses. For all significant main effects of covariates (tadpole stage or mass), we also conducted a Pearson's correlation to confirm the relationship between tadpole susceptibility to parasites, tadpole mass, and tadpole stage. For all significant correlations, to understand the degree to which tadpole stage or mass shapes tadpole susceptibility to trematodes, we conducted a regression analysis. All data in our experiment were analyzed using IBM SPSS software (Version 22, IBM, INC).

## 3 RESULTS

### 3.1 Effects of cold‐temperature regimes on tadpole development and mass

**Figure 2:**
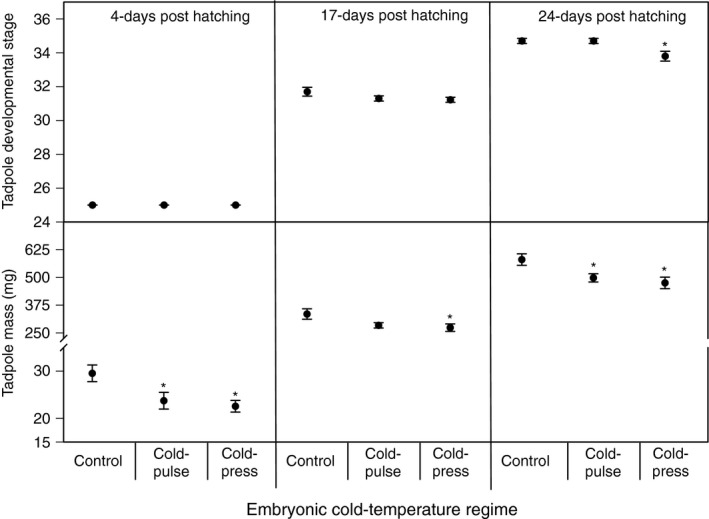
The effect of embryonic cold‐temperature regimes on tadpole development (Gosner Stage) and growth (mass) 4‐days, 17‐days, and 24‐days post‐hatching. The * symbol indicates a significant difference relative to the control treatment (*p* < 0.05)

For tadpoles that were 4‐days post‐hatching, we did not analyze the effect of embryonic cold‐temperature regimes on tadpole development because all tadpoles were at Gosner stage 25. Using analysis of variance (ANOVA), we found a significant univariate effect of embryonic cold‐temperature regime on tadpole mass at day 4 (*F*_2,27_ = 5.4, *p* = 0.01). Dunnett's pairwise comparison indicated that the cold‐press and the cold‐pulse treatments caused a significant decrease in tadpole mass relative to the control treatment (*p* = 0.009 and *p* = 0.031, respectively; Figure 2).

For tadpoles that were 17‐days post‐hatching, we did not analyze the effect of embryonic cold‐temperature regimes on tadpole development because all tadpoles were at stage 31. Using ANOVA, we found a marginally significant univariate effect of embryonic cold‐temperature regime on tadpole mass (*F*_2,27_ = 3.3, *p* = 0.052). Using Dunnett's pairwise comparisons, we found that the cold‐press but not the cold‐pulse treatments significantly decreased tadpole mass relative to the control treatment (*p* = 0.043 and *p* = 0.1, respectively; Figure 2).

For tadpoles that were 24‐days post‐hatching, we found a significant overall multivariate effect (MANOVA) of embryonic cold‐temperature regime on tadpole development and growth (Wilk's *λ* = 0.5, *F*_4,52_ = 5.2, *p* = 0.001). We found a significant effect of embryonic cold‐temperature regime on tadpole developmental stage 24‐days post‐hatching (*F*_2,27_ = 6.2, *p* = 0.006). The cold‐press treatment significantly reduced tadpole development compared to both the cold‐pulse (*p* = 0.014) and control treatments (*p* = 0.014; Figure 1). We also found a significant effect of embryonic cold‐temperature regime on tadpole mass 24‐days post‐hatching (*F*_2,27_ = 5.4, *p* = 0.01). Dunnett's pairwise comparisons indicated that both the cold‐press and the cold‐pulse treatments significantly decreased tadpole mass relative to the control treatment (*p* = 0.008 and *p* = 0.04, respectively; Figure 2).

### 3.2 Effects of cold‐temperature regimes on tadpole susceptibility to NaCl

**Figure 3:**
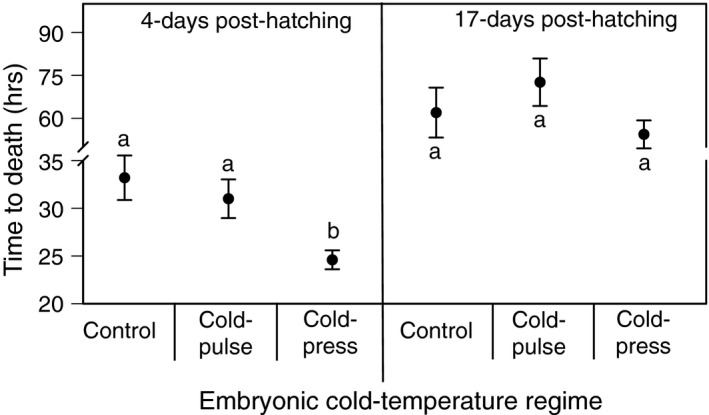
he effect of embryonic cold‐temperature regimes on tadpole susceptibility to NaCl (Time to death) at 4‐days and 17‐days post‐hatch. Treatments with different letters denote are significantly different from each other

For tadpoles not exposed to NaCl (control), we found 100% survival. For tadpoles 4‐days post‐hatching that were exposed to NaCl, we found a significant main effect of cold‐temperature regime on tadpole susceptibility to NaCl (Generalized linear model‐ GLM; *χ* ^2^ = 9.5; *p* = 0.009; Figure 3), but no effect on mass as a covariate (*χ* ^2^ = 0.12; *p* = 0.7). Sequential Bonferroni‐corrected analyses indicated that tadpoles from the cold‐press treatment were significantly more susceptible to NaCl compared to both the cold‐pulse (*p* = 0.025) and control treatments (*p* = 0.017).

In contrast, for tadpoles 17‐days post‐hatching that were exposed to NaCl, we found no significant main effect of cold‐temperature regime on tadpole susceptibility to NaCl (GLM; *χ* ^2^ = 4.2; *p* = 0.12; Figure 3) or on mass (*χ* ^2^ = 2.3; *p* = 0.13).

### 3.3 Effects of cold‐temperature regimes on tadpole susceptibility to parasites

**Figure 4:**
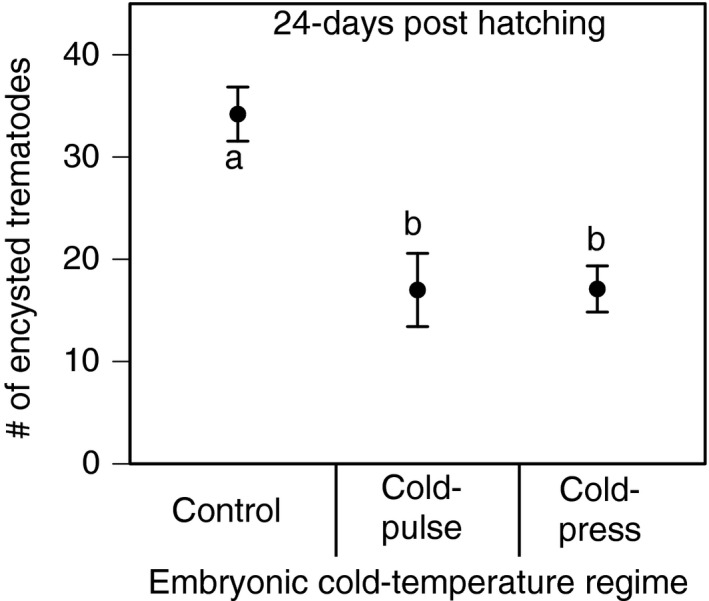
The effect of embryonic cold‐temperature regimes on tadpole susceptibility to parasites at 24‐days post‐hatch. Treatments with different letters denote are significantly different from each other

We found that all tadpoles in the parasite and control treatments survived until the end of the experiment and we confirmed that tadpoles from the control treatment were parasite‐free. Twenty‐four days post‐hatching, we found a significant main effect of cold‐temperature regime on tadpole susceptibility to parasites (GLM; *χ* ^2^ = 40.5; *p* < 0.001; Figure 4), on tadpole mass as a covariate (*χ* ^2^ = 120.5; *p* = 0.001), and on tadpole stage as a covariate (*χ* ^2^ = 6.0; *p* = 0.01). Sequential Bonferroni‐corrected analyses indicated that both the cold‐press and the cold‐pulse treatments significantly decreased tadpole susceptibility to parasites relative to the control treatment (for both *p* < 0.001; Figure 4). In contrast, the effects of the cold‐press treatment on tadpole susceptibility to parasites did not significantly differ from the effects of the cold‐pulse treatment (Figure 4).

**Figure 5:**
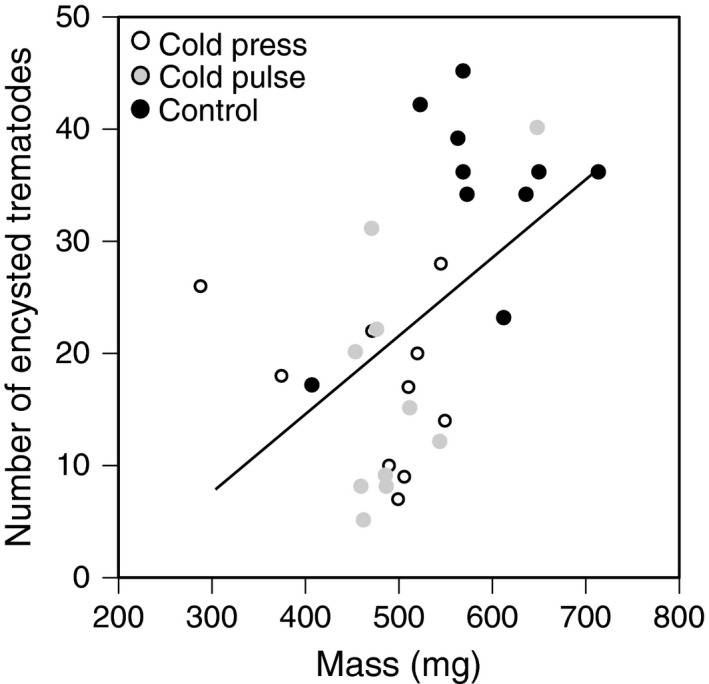
The relationship between tadpole mass and susceptibility to parasites 24‐days post‐hatch. Open circles represent tadpoles from the cold‐press treatment, gray circles represent tadpoles from the cold‐pulse treatment, and black circles represent tadpoles from the control treatment

We found a significant relationship between tadpole mass and trematode susceptibility to parasites using correlation analyses (*r* = 0.5, *p* = 0.005). However, despite a significant relationship between tadpole mass and tadpole stage (*r* = 0.6, *p* < 0.001), we found no relationship between tadpole stage and trematode susceptibility (*r* = 0.16, *p* = 0.40). The regression analysis demonstrated that for every unit increase in tadpole mass, we found a 0.5 (standardized beta coefficient) unit increase in tadpole susceptibility to parasites (*F* = 9.1, *p* = 0.005; Figure 5).

## 4 DISCUSSION

We investigated the effect of embryonic exposure to different cold‐temperature regimes (cold‐press and cold‐pulse) on wood frog tadpole development and growth, tolerance to NaCl, and susceptibility to parasites. Embryonic exposure to the cold‐press but not cold‐pulse treatment caused a delay in tadpole development relative to the control. The effect of the cold‐press treatment on development did not appear until 24‐days after hatching. Next, both cold‐press and cold‐pulse regimes caused a reduction in tadpole mass that persisted until the end of the study. Embryonic exposure to the cold‐press but not the cold‐pulse treatment caused an initial increase in tadpole susceptibility to NaCl, but these effects disappeared in older tadpoles. Finally, we found that both cold‐temperature regimes indirectly benefitted tadpoles by reducing their susceptibility to parasites. Collectively, we show that exposure to different cold‐temperature regimes differentially affected responses to common abiotic and biotic stressors later in life. These findings underscore the importance of considering the impact of cold temperatures in evaluating the consequences of global climate change.

Embryonic exposure to the cold‐press treatment but not cold‐pulse treatment resulted in a delay in tadpole development. Similarly, in a laboratory study, Orizaola, Dahl, and Laurila (2010) found that exposure to 4°C for 4‐days arrested early development and resulted in a longer period of time (2–3 days) between fertilization and metamorphosis compared to rearing at constant 15°C. In the field, Benard (2015), found that across several populations of wood frogs, on average every two days in advancement in breeding date, larval period increased by one day as a result of exposure to colder temperatures. Our study also suggests that the developmental effects associated with cold temperatures are not consistent across different cold‐temperature regimes (press vs. pulse). Indeed, across various study organisms and stressor types, even while holding the magnitude of stressor constant, consistent exposure to a stressor (i.e., presses) has been shown to be more detrimental than intermittent exposure to the stressor (i.e., pulses; Boone, Bridges, & Rothermel, 2001; Diamond, Klaine, & Butcher, 2006). In this study, we also found that the effect of the cold‐press treatment on development was not detected until 24‐days post‐hatching. This is consistent with the notion that early development is a highly conserved process where timing is largely controlled by intrinsic factors (Moss, 2007; Rougvie, 2005). Thus, the effects of cold temperatures can be overlooked if the effects are quantified early or at only a single time point. Collectively, due to climate shifts, incidences of forward phenological shifts and exposure to colder temperatures may become more common and future investigations should consider incorporating different cold‐temperature regimes and the potential for lag effects.

Both cold‐temperature regimes caused a consistent reduction in tadpole mass compared to the control. Contrary to our findings, past studies show that cold‐delayed tadpoles can induce compensatory growth responses (Murillo‐Rincón, Laurila, & Orizaola, 2017; Orizaola et al., 2010; Orizaola, Richter‐Boix, & Laurila, 2016). In our study, we did not observe the induction of compensatory growth responses, as the tadpoles exposed to both cold treatments were consistently smaller over the course of the study. Several factors may contribute to why our results differed compared to previous studies. First, the amphibian model (wood frog) used in our study differed than those used in other studies. Amphibians display species‐specific responses to stressors related to phylogeny (Hoverman, Gray, & Miller, 2010; Jones, Hammond, & Relyea, 2009; Searle et al., 2011). Thus, it is possible that our results may be due to species‐level variation in compensatory responses to cold temperature. Alternatively, in our study, we used only a single population of wood frogs (Lipo Pond). Populations commonly differ in their ability to respond to environmental conditions (i.e., plasticity; Hua et al., 2015a, 2015b); thus, it is possible that other populations of wood frogs may display compensatory growth in response to cold temperatures. Lastly, we only tracked tadpoles for 24‐days post‐hatching whereas (Orizaola et al., 2010) reared tadpoles to metamorphosis. Thus, it is possible that animals in our study may have displayed compensatory growth if the experiment were to be extended. Despite these differences in findings, climate change is expected to increase exposure of early spring‐breeding animals to cold temperatures and understanding factors shaping their ability to recover will become increasingly important.

Next, we investigated whether embryonic exposure to different cold‐temperature regimes altered tadpole susceptibility to NaCl. While both pulse and press treatments similarly delayed hatching (6 days) and similarly reduced tadpole mass relative to the control, we found that tadpoles exposed to cold‐press but not cold‐pulse treatments as embryos were more susceptible to lethal concentrations of NaCl at 4‐days post‐hatching. While the mechanisms responsible for these results are beyond the scope of this study, it is possible that the cold‐press environments, where embryos are exposed to prolonged constant cold temperatures, represents a more stressful condition compared to the cold‐pulse treatment, where embryos had opportunities to recover in warmer temperatures (Boone et al., 2001; Williams & Semlitsch, 2010). Interestingly, by 17‐days post‐hatching, we found that tadpoles reared in the cold‐press treatment as embryos were no longer more susceptible to NaCl compared to tadpoles reared in control conditions as embryos. However, consistent with past studies, tadpoles at 17‐days post‐hatching were significantly more tolerant to NaCl than tadpoles 4‐days post‐hatching (Ortiz‐Santaliestra, Marco, Fernández, & Lizana, 2006). This suggests that the effects of cold temperatures may only be detectable during more vulnerable early life stages. Collectively, our results demonstrate that tadpoles in the cold‐press treatment can recover the ability to respond to NaCl over time and highlights the importance of considering costs of exposure to cold temperatures across multiple cold‐temperature regimes and time points.

Finally, we investigated the influence of different cold‐temperature regimes on wood frog susceptibility to parasites. We hypothesized that tadpoles exposed to cold temperatures would exhibit higher infection loads because immune function has been demonstrated to be compromised in developmentally delayed tadpoles (Murillo‐Rincón et al., 2017). Similarly, Rohr and Raffel (2010) and Raffel et al. (2013) demonstrate that temperature variability increased host susceptibility to infection by the fungal parasite *Batrachochytrium dendrobatidis*. Contrary to our predictions, we found that relative to the control, tadpoles exposed to both cold‐temperature regimes were less susceptible to parasites. Our regression analysis demonstrates that host mass is positively related to infection load (Figure 5). This suggests that by causing a reduction in tadpole mass, both cold‐temperature regimes may have indirectly benefited tadpoles by reducing their susceptibility to parasites. Interestingly, past studies demonstrate that trematode susceptibility is not related to tadpole mass (Johnson & Hoverman, 2014) but is related to tadpole developmental stage with intermediate stages being the most susceptible (Holland et al., 2007; Raffel et al., 2011). In our study, though stage and mass are highly correlated (*r* = 0.6) we did not detect a relationship between trematode susceptibility and stage, though this may be due to the low variation in the developmental stages across treatments (average ± standard error; 34.4 ± 0.14). Nevertheless, these results suggest that future studies should consider whether cold‐temperature regime also influences other factors besides size (e.g., immune responses, behavior) to shape disease outcome. Global change is recognized as a major threat to the stability and biodiversity of host–parasite networks. The stability within these species interaction networks is key to maintaining ecosystem functioning, community stability and biodiversity (Carlson et al., 2017; Mougi & Kondoh, 2012; Strona & Lafferty, 2016). Our results demonstrate that shifts in temperature regimes can alter host–parasite interactions in unexpected indirect ways but considering different parasites that vary in host preference is critical for future studies.

Other considerations—In this study, we focus on wood frogs as our model because they are among the earliest breeding amphibians in North America. They are also the most likely species to both accelerate breeding in response to warming average temperatures and to face increased cold‐temperature variability (Benard, 2015; Gibbs & Breisch, 2001). However, to determine the generalizability of our findings, future studies should consider evaluating other species that also face increased cold‐temperature variability due to phenological accelerations in breeding (i.e., common frog; *Rana temporaria*; Phillimore, Hadfield, Jones, & Smithers, 2010). Additionally, given the wide geographic range of wood frogs, populations differ in average temperature increases and cold temperature variability. Because these differences may influence the generalizability of our findings, future studies should consider multiple populations. Next, increases in average temperature are expected to become more extreme over time (IPCC, 2014, 2018). This may eventually lead to accelerated breeding in other spring‐breeding amphibians that breed later than wood frogs (i.e., spring peepers; *Psuedacris crucifer*, American toads; *Anaxyrus americanus*) exposing them to novel variability in cold temperatures. Evaluating how these later spring‐breeding species respond is important to developing longer‐term generalizations regarding the dynamic ecological consequences of global climate change.

Finally, this study focuses specifically on evaluating the effect of heightened temperature variability. Accordingly, we manipulate embryonic exposure to different cold temperature regimes and not breeding time. Indeed, we manipulated temperatures variability while controlling for duration of exposure to cold temperatures by keeping the number of degree days (number of days with the temperature above the lower developmental threshold of 5.6°C; Herreid & Kinney, 1967) consistent at four days across all treatments. This is relevant because phenological shifts in breeding time in response to rising spring temperatures is a plastic trait that likely varies across natural populations. Populations that accelerate breeding time may be exposed to different cold‐temperature regimes or may be exposed to cold temperatures at different developmental stages compared to populations that do not accelerate breeding. To develop a broader understanding of how organisms respond to global shifts in average temperature and temperature variability, future work should consider also manipulating shifts in breeding times.

To sum, our work demonstrates that different cold‐temperature regimes (press vs. pulse) have unique effects across time emphasizing the importance of temperature variability in shaping populations impacted by global climate change. Additionally, while exposure to cold temperatures early in development may not always be directly lethal, it is important to still consider whether exposure to cold temperatures induces legacy effects that influence responses to other abiotic and biotic stressors later in life. Collectively, these results underscore the need to consider the impact of increased cold‐temperature variability to understand how global climate change is expected to influence ecological systems.

## CONFLICT OF INTEREST

None declared.

## AUTHOR CONTRIBUTIONS

MW and JH originally formulated the idea. All authors contributed to the design and performed the experiments. JH analyzed the data. All authors all contributed to writing the manuscript. The authors claim no conflicts of interest.

## Data Availability

A copy of the data can be found on the DRYAD international repository (https://doi.org/10.5061/dryad.dv4vk6c).

## References

[ece34957-bib-0001] Benard, M. F. (2015). Warmer winters reduce frog fecundity and shift breeding phenology, which consequently alters larval development and metamorphic timing. Global Change Biology, 21, 1058–1065. 10.1111/gcb.12720 25263760

[ece34957-bib-0002] Boone, M. D. , Bishop, C. A. , Boswell, L. A. , Brodman, R. D. , Burger, J. , Davidson, C. , … Weir, S. (2014). Pesticide regulation amid the Influence of Industry. BioScience, 64, 917–922. 10.1093/biosci/biu138

[ece34957-bib-0003] Boone, M. D. , Bridges, C. M. , & Rothermel, B. B. (2001). Growth and development of larval green frogs (*Rana clamitans*) exposed to multiple doses of an insecticide. Oecologia, 129, 518–524. 10.2307/4223115 24577691

[ece34957-bib-0004] Brooks, D. R. , & Hoberg, E. P. (2007). How will global climate change affect parasite–host assemblages? Trends in Parasitology, 23, 571–574. 10.1016/J.PT.2007.08.016 17962073

[ece34957-bib-0005] Brooks, D. R. , & Hoberg, E. P. (2015). Evolution in action: Climate change, biodiversity dynamics and emerging infectious disease. Philosophical Transactions of the Royal Society B: Biological Sciences, 370, 20130553 10.1098/rstb.2013.0553 PMC4342959 25688014

[ece34957-bib-0006] Brown, J. H. , Gillooly, J. F. , Allen, A. P. , Savage, V. M. , & West, G. B. (2004). Toward a metabolic theory of ecology. Ecology, 85, 1771–1789. 10.1890/03-9000

[ece34957-bib-0007] Buss, N. , & Hua, J. (2018). Parasite susceptibility in an amphibian host is modified by salinization and predators. Environmental Pollution, 236, 754–763. 10.1016/J.ENVPOL.2018.01.060 29455088

[ece34957-bib-0008] Cañedo‐Argüelles, M. , Hawkins, C. P. , Kefford, B. J. , Schafer, R. B. , Dyack, B. J. , Brucet, S. , … Timpano, A. J. (2016). Saving freshwater from salts. Science, 351, 914–916. 10.1126/science.aad3488 26917752

[ece34957-bib-0009] Carey, C. , & Alexander, M. A. (2003). Climate change and amphibian declines: Is there a link? Diversity and Distributions, 9, 111–121. 10.1046/j.1472-4642.2003.00011.x

[ece34957-bib-0010] Carlson, C. J. , Burgio, K. R. , Dougherty, E. R. , Phillips, A. J. , Bueno, V. M. , Clements, C. F. , … Getz, W. M. (2017). Parasite biodiversity faces extinction and redistribution in a changing climate. Science Advances, 3, e1602422 10.1126/sciadv.1602422 28913417 PMC5587099

[ece34957-bib-0011] Cohen, J. , Pfeiffer, K. , & Francis, J. A. (2018). Warm Arctic episodes linked with increased frequency of extreme winter weather in the United States. Nature Communications, 9, 869 10.1038/s41467-018-02992-9 PMC5849726 29535297

[ece34957-bib-0012] Cohen, J. M. , Venesky, M. D. , Sauer, E. L. , Civitello, D. J. , McMahon, T. A. , Roznik, E. A. , & Rohr, J. R. (2017). The thermal mismatch hypothesis explains host susceptibility to an emerging infectious disease. Ecology Letters, 20, 184–193. 10.1111/ele.12720 28111904

[ece34957-bib-0013] Collins, S. J. , & Russell, R. W. (2009). Toxicity of road salt to Nova Scotia amphibians. Environmental Pollution, 157, 320–324. 10.1016/J.ENVPOL.2008.06.032 18684543

[ece34957-bib-0014] Conant, R. , & Collins, J. T. (1998). A field guide to reptiles & amphibians: Eastern and central North America. Boston, MA: Houghton Mifflin.

[ece34957-bib-0015] Daszak, P. , Cunningham, A. A. , & Hyatt, A. D. (2000). Emerging infectious diseases of wildlife–threats to biodiversity and human health. Science, 287, 443–449. 10.1126/science.287.5452.443 10642539

[ece34957-bib-0016] Diamond, J. M. , Klaine, S. J. , & Butcher, J. B. (2006). Implications of pulsed chemical exposures for aquatic life criteria and wastewater permit limits. Environmental Science and Technology, 40, 5132–5138. 10.1021/ES0604358 16955918

[ece34957-bib-0017] Dietz, M. E. , Angel, D. R. , Robbins, G. A. , & McNaboe, L. A. (2016). Permeable asphalt: A new tool to reduce road salt contamination of groundwater in urban areas. Groundwater, 55, 237–243. 10.1111/gwat.12454 27576128

[ece34957-bib-0018] Dugan, H. A. , Bartlett, S. L. , Burke, S. M. , Doubek, J. P. , Krivak‐Tetley, F. E. , Skaff, N. K. , Weathers, K. C. (2017). Salting our freshwater lakes. Proceedings of the National Academy of Sciences of the United States of America, 114, 4453–4458. 10.1073/pnas.1620211114 28396392 PMC5410852

[ece34957-bib-0019] Esteban, J. G. , & Muñoz‐Antoli, C. (2009). Echinostomes: Systematics and life cycles In Bernard F., & Rafael T. (Eds.), The biology of Echinostomes (pp. 1–34). New York, NY: Springer.

[ece34957-bib-0020] Findlay, S. E. G. , & Kelly, V. R. (2011). Emerging indirect and long‐term road salt effects on ecosystems. Annals of the New York Academy of Sciences, 1223, 58–68. 10.1111/j.1749-6632.2010.05942.x 21449965

[ece34957-bib-0021] Frisbie, M. P. , Costanzo, J. P. , & Lee, R. E. (2000). Physiological and ecological aspects of low‐temperature tolerance in embryos of the wood frog, *Rana sylvatica* . Canadian Journal of Zoology, 78, 1032–1041. 10.1139/z00-022

[ece34957-bib-0022] Gibbs, J. P. , & Breisch, A. R. (2001). Climate warming and calling phenology of frogs near Ithaca, New York, 1900–1999. Conservation Biology, 15, 1175–1178. 10.1046/j.1523-1739.2001.0150041175.x

[ece34957-bib-0023] Gosner, K. L. (1960). A simplified table for staging anuran embryos and larvae with notes on identification. Herpetologica, 16, 183–190.

[ece34957-bib-0024] Herbert, E. R. , Boon, P. , Burgin, A. J. , Neubauer, S. C. , Franklin, R. B. , Ardón, M. , … Gell, P. (2015). A global perspective on wetland salinization: ecological consequences of a growing threat to freshwater wetlands. Ecosphere, 6(10), art206 10.1890/ES14-00534.1

[ece34957-bib-0025] Herreid, C. F. , & Kinney, S. (1967). Temperature and development of the wood frog, *Rana Sylvatica*, in Alaska. Ecology, 48, 579–590. 10.2307/1936502

[ece34957-bib-0026] Hilty, J. , & Merenlender, A. (2000). Faunal indicator taxa selection for monitoring ecosystem health. Biological Conservation, 92, 185–197. 10.1016/S0006-3207(99)00052-X

[ece34957-bib-0027] Holland, M. P. , Skelly, D. K. , Kashgarian, M. , Bolden, S. R. , Harrison, L. M. , & Cappello, M. (2007). Echinostome infection in green frogs (*Rana clamitans*) is stage and age dependent. Journal of Zoology, 271, 455–462. 10.1111/j.1469-7998.2006.00229.x

[ece34957-bib-0028] Hoverman, J. , Gray, M. , & Miller, D. (2010). Anuran susceptibilities to ranaviruses: Role of species identity, exposure route, and a novel virus isolate. Diseases of Aquatic Organisms, 89, 97–107. 10.3354/dao02200 20402227

[ece34957-bib-0029] Howard, R. D. (1980). Mating behavior and mating success in wood frogs, *Rana sylvatica* . Animal Behaviour, 28, 705–716.

[ece34957-bib-0030] Hua, J. , Buss, N. , Kim, J. , Orlofske, S. A. , & Hoverman, J. T. (2016). Population‐specific toxicity of six insecticides to the trematode Echinoparyphium sp. Parasitology, 143, 542–550. 10.1017/S0031182015001894 26928351

[ece34957-bib-0031] Hua, J. , Jones, D. K. , Mattes, B. M. , Cothran, R. D. , Relyea, R. A. , & Hoverman, J. T. (2015a). Evolved pesticide tolerance in amphibians: Predicting mechanisms based on pesticide novelty and mode of action. Environmental Pollution, 206, 56–63. 10.1016/j.envpol.2015.06.030 26142751

[ece34957-bib-0032] Hua, J. , Jones, D. K. , Mattes, B. M. , Cothran, R. D. , Relyea, R. A. , & Hoverman, J. T. (2015b). The contribution of phenotypic plasticity to the evolution of insecticide tolerance in amphibian populations. Evolutionary Applications, 8, 586–596. 10.1111/eva.12267 26136824 PMC4479514

[ece34957-bib-0034] Inouye, D. W. (2008). Effects of climate change on phenology, frost damage, and floral abundance of montane wildflowers. Ecology, 89, 353–362. 10.1890/06-2128.1 18409425

[ece34957-bib-0035] IPCC (2014). Climate Change 2014: Synthesis report. In Core Writing Team, Pachauri R. K., & Meyer L. A. (Eds.), Contribution of Working Groups I, II and III to the Fifth Assessment Report of the Intergovernmental Panel on Climate Change. Geneva, Switzerland: IPCC.

[ece34957-bib-0036] IPCC (2018). Summary for policymakers In Global warming of 1.5°C. An IPCC Special Report on the impacts of global warming of 1.5°C above pre‐industrial levels and related global greenhouse gas emission pathways, in the context of strengthening the global response to the threat of climate change, sustainable development, and efforts to eradicate poverty. Geneva, Switzerland: World Meteorological Organization.

[ece34957-bib-0037] Johnson, P. T. J. , & Hoverman, J. T. (2014). Heterogeneous hosts: How variation in host size, behaviour and immunity affects parasite aggregation. Journal of Animal Ecology, 83, 1103–1112. 10.1111/1365-2656.12215 24548254

[ece34957-bib-0038] Johnson, P. T. J. , & McKenzie, V. J. (2009). Effects of environmental change on helminth infections in amphibians: Exploring the emergence of Ribeiroia and Echinostoma infections in North America In Bernard F., & Rafael T. (Eds.), The biology of Echinostomes (pp. 249–280). New York, NY: Springer.

[ece34957-bib-0039] Johnson, P. T. J. , Rohr, J. R. , Hoverman, J. T. , Kellermanns, E. , Bowerman, J. , & Lunde, K. B. (2012). Living fast and dying of infection: Host life history drives interspecific variation in infection and disease risk. Ecology Letters, 15, 235–242. 10.1111/j.1461-0248.2011.01730.x 22221837

[ece34957-bib-0040] Jones, D. K. , Hammond, J. I. , & Relyea, R. A. (2009). Very highly toxic effects of endosulfan across nine species of tadpoles: Lag effects and family‐level sensitivity. Environmental Toxicology and Chemistry, 28, 1939–1945. 10.1897/09-033.1 19358624

[ece34957-bib-0041] Karraker, N. E. , Gibbs, J. P. , & Vonesh, J. R. (2008). Impacts of road deicing salt on the demography of vernal pool‐breeding amphibians. Ecological Applications, 18, 724–734. 10.1890/07-1644.1 18488630

[ece34957-bib-0042] Kaushal, S. S. , Groffman, P. M. , Likens, G. E. , Belt, K. T. , Stack, W. P. , Kelly, V. R. , … Fisher, G. T. (2005). Increased salinization of fresh water in the northeastern United States. Proceedings of the National Academy of Sciences of the United States of America, 102, 13517–13520. 10.1073/pnas.0506414102 16157871 PMC1224654

[ece34957-bib-0043] Kelly, V. , Findlay, S. , Schlesinger, W. , Menking, K. , & Chatrchyan, A. (2010). Road salt: Moving toward the solution. The Cary Institute of Ecosystem Studies.

[ece34957-bib-0044] Kostadinova, A. , & Gibson, D. I. (2000). The systematics of the echinostomes In Fried B. (Ed.), Echinostomes as experimental models for biological research (pp. 31–57). New York, NY: Springer.

[ece34957-bib-0045] Kretschmer, M. , Coumou, D. , Agel, L. , Barlow, M. , Tziperman, E. , & Cohen, J. (2017). More‐persistent weak Stratospheric polar vortex states linked to cold extremes. Bulletin of the American Meteorological Society, 99, 49–60. 10.1175/BAMS-D-16-0259.1

[ece34957-bib-0046] Kutz, S. J. , Hoberg, E. P. , Polley, L. , & Jenkins, E. J. (2005). Global warming is changing the dynamics of Arctic host‐parasite systems. Proceedings of the Royal Society B: Biological Sciences, 272, 2571–2576. 10.1098/rspb.2005.3285 PMC1559981 16321777

[ece34957-bib-0047] Labaude, S. , Moret, Y. , Cézilly, F. , Reuland, C. , & Rigaud, T. (2017). Variation in the immune state of Gammarus pulex (Crustacea, Amphipoda) according to temperature: Are extreme temperatures a stress? Developmental & Comparative Immunology, 76, 25–33. 10.1016/j.dci.2017.05.013 28522173

[ece34957-bib-0048] Li, Y. , Cohen, J. M. , & Rohr, J. R. (2013). Review and synthesis of the effects of climate change on amphibians. Integrative Zoology, 8, 145–161. 10.1111/1749-4877.12001 23731811

[ece34957-bib-0050] Maldonado, A. , & Lanfredi, R. M. (2009). Echinostomes in the wild In Bernard F., & Rafael T. (Eds.), The biology of Echinostomes: From the molecule to the community (pp. 129–145). New York, NY: Springer.

[ece34957-bib-0051] Martin, T. R. , & Conn, D. B. (1990). The pathogenicity, localization, and cyst structure of echinostomatid metacercariae (Trematoda) infecting the kidneys of the frogs *Rana clamitans* and *Rana pipiens* . The Journal of Parasitology, 76, 414–419. 10.2307/3282677 2352071

[ece34957-bib-0052] McCullagh, P. , & Nelder, J. A. (1989). Generalised linear models II. London, UK: Chapman and Hall.

[ece34957-bib-0054] Moss, E. G. (2007). Heterochronic genes and the nature of developmental time. Current Biology, 17, R425–R434. 10.1016/J.CUB.2007.03.043 17550772

[ece34957-bib-0055] Mougi, A. , & Kondoh, M. (2012). Diversity of interaction types and ecological community stability. Science, 337, 349–351. 10.1126/science.1220529 22822151

[ece34957-bib-0056] Murillo‐Rincón, A. P. , Laurila, A. , & Orizaola, G. (2017). Compensating for delayed hatching reduces offspring immune response and increases life‐history costs. Oikos, 126, 565–571. 10.1111/oik.04014

[ece34957-bib-0057] Newman, M. C. (2006). Fundamentals of ecotoxicology: the science of pollution. Boca Raton, FL: CRC Press.

[ece34957-bib-0058] Orizaola, G. , Dahl, E. , & Laurila, A. (2010). Compensating for delayed hatching across consecutive life‐history stages in an amphibian. Oikos, 119, 980–987. 10.1111/j.1600-0706.2009.17956.x

[ece34957-bib-0059] Orizaola, G. , Richter‐Boix, A. , & Laurila, A. (2016). Transgenerational effects and impact of compensatory responses to changes in breeding phenology on antipredator defenses. Ecology, 97, 2470–2478. 10.1002/ecy.1464 27859081

[ece34957-bib-0060] Ortiz‐Santaliestra, M. E. , Marco, A. , Fernández, M. J. , & Lizana, M. (2006). Influence of developmental stage on sensitivity to ammonium nitrate of aquatic stages of amphibians. Environmental Toxicology and Chemistry, 25, 105–111. 10.1897/05-023R.1 16494230

[ece34957-bib-0061] Parmesan, C. , & Yohe, G. (2003). A globally coherent fingerprint of climate change impacts across natural systems. Nature, 421, 37–42. 10.1038/nature01286 12511946

[ece34957-bib-0062] Patra, R. W. , Chapman, J. C. , Lim, R. P. , Gehrke, P. C. , & Sunderam, R. M. (2015). Interactions between water temperature and contaminant toxicity to freshwater fish. Environmental Toxicology and Chemistry, 34, 1809–1817. 10.1002/etc.2990 26033197

[ece34957-bib-0063] Phillimore, A. B. , Hadfield, J. D. , Jones, O. R. , & Smithers, R. J. (2010). Differences in spawning date between populations of common frog reveal local adaptation. Proceedings of the National Academy of Sciences of the United States of America, 107, 8292–8297. 10.1073/pnas.0913792107 20404185 PMC2889515

[ece34957-bib-0064] Podolsky, R. D. , & Emlet, R. B. (1993). Separating the effects of temperature and viscosity on swimming and water movement by sand dollar larvae (Dendraster Excentricus). Journal of Experimental Biology, 221, 207–221.

[ece34957-bib-0065] Pollister, A. W. , & Moore, J. A. (1937). Tables for the normal development of *Rana sylvatica* . The Anatomical Record, 68, 489–496. 10.1002/ar.1090680410

[ece34957-bib-0066] Raffel, T. R. , Lloyd‐Smith, J. O. , Sessions, S. K. , Stanley, K. , Hudson, P. J. , & Rohr, J. R. (2011). Does the early frog catch the worm? Disentangling potential drivers of a parasite age–intensity relationship in tadpoles. Oecologia, 165, 1031–1042. 10.1007/s00442-010-1776-0 20852894 PMC3057004

[ece34957-bib-0067] Raffel, T. R. , Rohr, J. R. , Kiesecker, J. M. , & Hudson, P. J. (2006). Negative effects of changing temperature on amphibian immunity under field conditions. Functional Ecology, 20, 819–828. 10.1111/j.1365-2435.2006.01159.x

[ece34957-bib-0068] Raffel, T. R. , Romansic, J. M. , Halstead, N. T. , McMahon, T. A. , Venesky, M. D. , & Rohr, J. R. (2013). Disease and thermal acclimation in a more variable and unpredictable climate. Nature Climate Change, 3, 146–151. 10.1038/nclimate1659

[ece34957-bib-0069] Rohr, J. R. , & Raffel, T. R. (2010). Linking global climate and temperature variability to widespread amphibian declines putatively caused by disease. Proceedings of the National Academy of Sciences of the United States of America, 107, 8269–8274. 10.1073/pnas.0912883107 20404180 PMC2889522

[ece34957-bib-0070] Rohr, J. R. , Raffel, T. R. , & Hall, C. A. (2010). Developmental variation in resistance and tolerance in a multi‐host‐parasite system. Functional Ecology, 24, 1110–1121. 10.1111/j.1365-2435.2010.01709.x

[ece34957-bib-0071] Rougvie, A. E. (2005). Intrinsic and extrinsic regulators of developmental timing: From miRNAs to nutritional cues. Development, 132, 3787–3798. 10.1242/dev.01972 16100088

[ece34957-bib-0072] Rumschlag, S. L. , Boone, M. D. , & Fellers, G. (2014). The effects of the amphibian chytrid fungus, insecticide exposure, and temperature on larval anuran development and survival. Environmental Toxicology and Chemistry, 33, 2545–2550. 10.1002/etc.2707 25098758

[ece34957-bib-0073] Sanzo, D. , & Hecnar, S. J. (2006). Effects of road de‐icing salt (NaCl) on larval wood frogs (Rana sylvatica). Environmental Pollution, 140, 247–256. 10.1016/J.ENVPOL.2005.07.013 16159689

[ece34957-bib-0074] Searle, C. L. , Gervasi, S. S. , Hua, J. , Hammond, J. I. , Relyea, R. A. , Olson, D. H. , & Blaustein, A. R. (2011). Differential host susceptibility to Batrachochytrium dendrobatidis, an emerging amphibian pathogen. Conservation Biology, 25, 965–974. 10.1111/j.1523-1739.2011.01708.x 21732979

[ece34957-bib-0075] Strona, G. , & Lafferty, K. D. (2016). Environmental change makes robust ecological networks fragile. Nature Communications, 7, 12462 10.1038/ncomms12462 PMC4987532 27511722

[ece34957-bib-0076] Thackeray, S. J. , Henrys, P. A. , Hemming, D. , Bell, J. R. , Botham, M. S. , Burthe, S. , Wanless, S. (2016). Phenological sensitivity to climate across taxa and trophic levels. Nature, 535, 241–245. 10.1038/nature18608 27362222

[ece34957-bib-0077] Visser, M. E. , & Both, C. (2005). Shifts in phenology due to global climate change: The need for a yardstick. Proceedings of the Royal Society B: Biological Sciences, 272, 2561–2569. 10.1098/rspb.2005.3356 PMC1559974 16321776

[ece34957-bib-0078] Waldman, B. (1982). Adaptive significance of communal oviposition in wood frogs (*Rana sylvatica*). Behavioral Ecology and Sociobiology, 10, 169–174. 10.2307/4599479

[ece34957-bib-0079] Walther, G.‐R. , Post, E. , Convey, P. , Menzel, A. , Parmesan, C. , Beebee, T. J. C. , … Bairlein, F. (2002). Ecological responses to recent climate change. Nature, 416, 389–395. 10.1038/416389a 11919621

[ece34957-bib-0080] Weatherley, A. H. , & Gill, H. S. (1983). Protein, lipid, water and caloric contents of immature rainbow trout, Salmo gairdneri Richardson, growing at different rates. Journal of Fish Biology, 23, 653–673. 10.1111/j.1095-8649.1983.tb02944.x

[ece34957-bib-0081] Williams, B. K. , & Semlitsch, R. D. (2010). Larval responses of three Midwestern Anurans to chronic, low‐dose exposures of four herbicides. Archives of Environmental Contamination and Toxicology, 58, 819–827. 10.1007/s00244-009-9390-z 19768486

[ece34957-bib-0082] Yee, E. H. , & Murray, S. N. (2004). Effects of temperature on activity, food consumption rates, and gut passage times of seaweed‐eating Tegula species (Trochidae) from California. Marine Biology, 145, 895–903. 10.1007/s00227-004-1379-6

